# Retraction Note: Exosomal microRNA-32-5p induces multidrug resistance in hepatocellular carcinoma via the PI3K/Akt pathway

**DOI:** 10.1186/s13046-023-02761-7

**Published:** 2023-07-19

**Authors:** Xiao Fu, Mengjie Liu, Shengyang Qu, Jiequn Ma, Yamin Zhang, Tingting Shi, Hongqing Wen, Yujuan Yang, Shuhong Wang, Jing Wang, Kejun Nan, Yu Yao, Tao Tian

**Affiliations:** 1 Department of Medical Oncology, The First Affiliated Hospital of Xi’an Jiaotong University, No. 277 Yanta West Road, Xi’an, 710061 Shaanxi People’s Republic of China; 2 Department of Respiratory, Third Hospital of Xi’an, Xi’an, 710018 Shaanxi People’s Republic of China; 3 The Third Department of Cardiology, Shaanxi Provincial People’s Hospital, Xi’an, 710068 Shaanxi province People’s Republic of China

**Retraction Note: ***J Exp Clin Cancer Res* 37, 52 (2018)

10.1186/s13046-018-0677-7

The Editor-in-Chief has retracted this article. After publication, concerns were raised regarding highly similar images in the present figures, specifically:

- The Transwell assay images in Fig. 6e and f, and 7i appear to contain multiple overlapping panels;
- A number of images in Fig. 6e appear highly similar to those in Figs. 4d and 5d in the authors’ earlier article [1].
- Figure 7h middle panel p-Akt lanes 3 and 4 appear highly similar to Fig. 7k right panel N-Cad lanes 1 and 2.
- Figure 7h left and right panel b-actin blots appear highly similar to those in Fig. 7k, but the middle panel blots are different.

Additionally, the study used Bel7402 and Bel/5-FU cell lines, which have both been reported to be HeLa derivatives. Therefore, these cells are not suitable models for hepatocellular carcinoma.

The authors have provided the raw data from the Transwell, immunohistochemistry and western blot assays. However, further checks by the publisher found labelling errors in the original western blot data that may have affected the results and conclusions of the article. The Editor-in-Chief therefore no longer has confidence in the presented data.

Corresponding Author Tao Tian has stated on behalf of all co-authors that they do not agree to this retraction.
